# Inhibitory Effect of Natural Anti-Inflammatory Compounds on Cytokines Released by Chronic Venous Disease Patient-Derived Endothelial Cells

**DOI:** 10.1155/2013/423407

**Published:** 2013-12-31

**Authors:** Veronica Tisato, Giorgio Zauli, Erika Rimondi, Sergio Gianesini, Laura Brunelli, Erica Menegatti, Paolo Zamboni, Paola Secchiero

**Affiliations:** ^1^Department of Morphology, Surgery and Experimental Medicine and LTTA Centre, University of Ferrara, Via Fossato di Mortara 66, 44100 Ferrara, Italy; ^2^Institute for Maternal and Child Health, IRCCS Burlo Garofolo, Via dell'Istria 65/01, 34137 Trieste, Italy; ^3^Department of Life Sciences, University of Trieste, Via Manzoni 16, 34137 Trieste, Italy; ^4^Vascular Disease Center, University of Ferrara, Via Aldo Moro 8, 44124 Ferrara, Italy

## Abstract

Large vein endothelium plays important roles in clinical diseases such as chronic venous disease (CVD) and thrombosis; thus to characterize CVD vein endothelial cells (VEC) has a strategic role in identifying specific therapeutic targets. On these bases we evaluated the effect of the natural anti-inflammatory compounds **α**-Lipoic acid and Ginkgoselect phytosome on cytokines/chemokines released by CVD patient-derived VEC. For this purpose, we characterized the levels of a panel of cytokines/chemokines (*n* = 31) in CVD patients' plasma compared to healthy controls and their release by VEC purified from the same patients, in unstimulated and TNF-**α** stimulated conditions. Among the cytokines/chemokines released by VEC, which recapitulated the systemic profile (IL-8, TNF-**α**, GM-CSF, INF-**α**2, G-CSF, MIP-1**β**, VEGF, EGF, Eotaxin, MCP-1, CXCL10, PDGF, and RANTES), we identified those targeted by *ex vivo* treatment with **α**-Lipoic acid and/or Ginkgoselect phytosome (GM-CSF, G-CSF, CXCL10, PDGF, and RANTES). Finally, by investigating the intracellular pathways involved in promoting the VEC release of cytokines/chemokines, which are targeted by natural anti-inflammatory compounds, we documented that **α**-Lipoic acid significantly counteracted TNF-**α**-induced NF-*κ*B and p38/MAPK activation while the effects of *Ginkgo biloba* appeared to be predominantly mediated by Akt. Our *data* provide new insights into the molecular mechanisms of CVD pathogenesis, highlighting new potential therapeutic targets.

## 1. Introduction

Although *α*-Lipoic acid has been firstly explored in the light of its natural antioxidant properties and its ability to induce apoptosis in several tumour cell lines [1, 2], the therapeutic implications related to its clinical use have been extensively reviewed underlying overall beneficial effects on several pathological conditions such as diabetes mellitus and atherosclerosis [3, 4]. Among the putative cellular therapeutic targets of *α*-Lipoic acid, also the endothelial compartment has been considered and some reports suggested a protective effect of *α*-Lipoic acid on endothelial cell damage [5]. In the same fashion, *Ginkgo biloba* extract derivatives have been evaluated in “standard” endothelial cell models (such as human umbilical vein endothelial cells, HUVEC) as vascular protective agents to attenuate oxidative stress damage [6].

Endothelial cells derived from different anatomical districts are heterogeneous in terms of the pattern of antigens expression, secreted molecules, and immunological properties and therefore common *in vitro* endothelial models, such as HUVEC, cannot properly recapitulate the *in vivo* features of pathological endothelium. In this light, the responses of large vein endothelium are thought to play important roles in clinical diseases, such as chronic venous disease (CVD) and thrombosis [7, 8]. The endothelium actively reacts in response to changes of the local environment by expressing and releasing specific cytokines, chemokines, and soluble chemical mediators that, in turn, play a role in the pathophysiology of CVD [9–12]. The therapeutic options for CVD patients range from conservative therapies, minimally invasive approaches, and surgical treatments; nevertheless CVD remains a disease with a high recurrence rate [13].

In the present study, we have addressed if the natural compounds *α*-Lipoic acid and *Ginkgo biloba* might have a role in treating CVD through a direct effect on the pathological endothelium, by using vein endothelial cells (VEC) isolated from patients at different stages of CVD. In particular, we have evaluated the anti-inflammatory effect of the natural compounds *α*-Lipoic acid and Ginkgoselect phytosome on cytokines/chemokines released by CVD patient-derived VEC. For this purpose, we first characterized the systemic levels of a wide panel of cytokines/chemokines (*n* = 31) in the plasma of patients affected by CVD with respect to normal healthy controls and the release of these cytokines/chemokines by VEC purified from the same CVD patients, in unstimulated and TNF-*α*-stimulated conditions. Finally, we investigated the intracellular signal transduction pathways involved in promoting the VEC release of cytokines/chemokines, which are targeted by natural anti-inflammatory compounds. Overall, the data reported in the present study provide new insights into the molecular mechanisms of CVD pathogenesis, highlighting new potential therapeutic targets.

## 2. Materials and Methods

### 2.1. Recruitment of Patients and Samples Collection

The main demographic/clinical characteristics of CVD patients enrolled in this study are reported in (see Supplementary Table 1 in Supplementary Material available online at http://dx.doi.org/10.1155/2013/423407). Forearm blood samples of patients affected by primary CVD with superficial venous reflux (C2-4EpAsPr following the CEAP classification) were collected in the presence of sodium citrate before varicose veins surgery. Blood samples were immediately centrifuged for plasma isolation that were stored at −80°C in single-use aliquots. Plasma samples collected from healthy subjects were used as controls. The surgical specimens were collected during surgery for conservative and hemodynamic treatment of venous insufficiency in ambulatory care. Highly purified VEC cultures obtained from CVD patients were isolated and characterized for morphological and phenotypic properties, as previously described [14]. The procedures followed were in accordance with the Declaration of Helsinki, approved by the institutional review board (University Hospital of Ferrara) and all participant subjects gave written informed consent.

### 2.2. Endothelial Cell Cultures and Treatments

Patient-derived endothelial cells were used within passages 3 to 7. Cells were grown on EGM2 medium (Lonza, Walkersville, MD) with 2% FBS and full supplements (EGM2 Bullet kit, Lonza) in 5 *μ*g/cm^2^ fibronectin precoated tissue culture plates (BD, Becton Dickinson, San Josè, CA), as described previously [15].

For endothelial cell treatments, the following reagents have been used: recombinant tumor necrosis factor (TNF)-*α* (R&D Systems, Minneapolis, MN), Ginkgoselect phytosome, and *α*-Lipoic acid (kindly provided by LABOREST S.p.A., Milano, Italy) were dissolved in distilled water and in dimethylsulfoxide (DMSO, Sigma, St. Louis, MO), respectively, and at a final concentration such that the DMSO did not exceed 0.1% in the cell culture media. The optimal concentrations of TNF-*α* (5 ng/mL), Ginkgoselect phytosome, and *α*-Lipoic acid (both used at 100 *μ*g/mL) were determined in preliminary dose-response experiments. Ginkgoselect phytosome and *α*-Lipoic acid were added to the cells 1 hour before treatment with TNF-*α*. In selected experiments, cells were preincubated for 1 hour with pharmacologic inhibitors of ERK1/2 (PD98059; 50 *μ*M; Calbiochem, La Jolla, CA), p38/MAPK (SB203580; 10 *μ*M; Calbiochem), NF-*κ*B (Parthenolide; 10 *μ*M; Enzo Life Science, Exeter, UK), and Akt (MK-2206; 5 *μ*M; BioVision Incorporated, Milpitas, CA) pathways prior to the addition of TNF-*α*. Cell supernatants were collected from endothelial cultures and frozen at −80°C as described [16] until the cytokine analyses were performed.

### 2.3. Assessment of Cell Viability and Cell Surface Inflammatory Markers

Cell viability was monitored by light microscopic analysis of the cell monolayers after hematoxylin-eosin staining or by quantitative examination after detachment of the monolayers by means of Trypan blue dye exclusion, as described [17, 18]. The profiles of expression of the inflammatory markers ICAM-1 and VCAM-1 were analyzed by flow cytometry as previously described [19]. In brief, cells were detached with trypsin-EDTA and washed and 5 × 10^5^ cells were resuspended in 200 *μ*L of PBS containing 1% BSA (Sigma-Aldrich) and incubated for 30 minutes at 4°C with the following monoclonal antibodies (mAb): FITC-conjugated anti-ICAM-1 (R&D, Clone BBIG-I1) or FITC-conjugated anti-VCAM-1 (R&D, Clone BBIG-V3). Nonspecific fluorescence was assessed by incubation with isotype-matched conjugated mAb [20].

### 2.4. Analysis of Cytokines and Chemokines in Plasma and Cell Culture Supernatants

Culture supernatant samples and plasma of patients were frozen and thawed only once before performing the MILLIPLEX MAP Human Cytokine/Chemokine Panel (Merck Millipore, Billerica, MA), a bead-based multiplex immunoassay, which allows the simultaneous quantification of the following 29 human cytokines: EGF, IL-1*β*, IL-1 receptor antagonist (ra), IL-1*α*, IL-2, IL-3, IL-4, IL-5, IL-6, IL-7, IL-8, IL-10, IL-12(p40), IL-12(p70), IL-13, IL-15, 1L-17A, EOTAXIN, G-CSF, GM-CSF, IFN-*α*2, IFN-*γ*, CXCL10, MCP-1, MIP-1*α*, MIP-1*β*, TNF-*α*, TNF-*β*, and VEGF. Moreover, a custom-made MILLIPLEX MAP Human Cytokine/Chemokine Magnetic Bead Panel (Merck Millipore) was used to quantify the cytokines PDGF-AB/BB and RANTES. Culture supernatant samples were processed following the manufacturer's recommended protocols and read on a MAGPIX instrument equipped with the MILLIPLEX-Analyst Software using a five-parameter nonlinear regression formula to compute sample concentrations from the standard curves.

### 2.5. Phosphoproteins Profile and Western Blot in Endothelial Cells

The MILLIPLEX MAP Human Multi-Pathway 9-plex Magnetic Bead Signalling kit phosphoprotein was used to detect changes in phosphorylated ERK/MAP kinase 1/2 (Thr185/Tyr187), Akt (Ser473), STAT3 (Ser727), JNK (Thr183/Tyr185), p70 S6 kinase (Thr412), NF-*κ*B (Ser536), STAT5A/B (Tyr694/699), CREB (Ser133), and p38 (Thr180/Tyr182) in endothelial cell lysates using the Luminex system, according to the manufacturer's instructions. Briefly, endothelial cells were plated in 100 mm dishes, grown at subconfluence, and subjected to partial FCS reduction (to 0.5%) for 18 hours before the addition of treatments. Cells were harvested in MILLIPLEX MAP lysis buffer in the presence of Protease Inhibitor Cocktail Set III (Calbiochem). Each lysate (20 *μ*g total protein) was diluted in MILLIPLEX MAP Assay Buffer 2, incubated at 4°C overnight, and analyzed according to the manufacturer's instructions. The Median Fluorescence Intensity (MFI) was measured with the Luminex System.

To validate the MILLIPLEX MAP human multipathway data, we have performed Western blot analyses as previously described [21, 22]. Briefly, cells were harvested in lysis buffer containing 1% Triton X-100, 150 mM NaCl, 50 mM Tris-HCl pH 6.8, and protease inhibitors (protease inhibitor cocktail; Roche Diagnostics). Protein determination was performed by Bradford assay (Bio-Rad, Richmond, CA). Equal amounts of protein (50 *μ*g) for each sample were migrated in acrylamide gels, blotted onto nitrocellulose filters, and probed with the following antibodies for the phosphorylated forms of ERK1/2, p38/MAPK, and IkB and for the respective total protein kinase content for verifying loading evenness: anti-phospho-p44/42 MAPK (Erk1/2) and anti-p44/42 MAPK (Erk1/2) (both from Cell Signalling Technology, Danvers, MA); anti-phospho-p38/MAPKinase and anti-p38/MAPKinase (both from Cell Signaling Technology, Beverly, MA); anti-phospho-Akt and anti-Akt (both from Merck Millipore); anti-IkB (from Santa Cruz Biotechnology, Santa Cruz, CA) and antitubulin (Sigma-Aldrich). After incubation with peroxidase-conjugated anti-mouse and anti-rabbit IgG, specific reactions were revealed with the ECL Lightning detection kit (Perkin Elmer, Boston, MA). Densitometry values were estimated by the ImageQuant TL software (GE Healthcare Bio-Sciences AB, Uppsala, Sweden). Multiple film exposures were used to verify the linearity of the samples analyzed and avoid saturation of the film.

### 2.6. Statistical Analysis

Descriptive statistics were calculated. For each set of experiments, values were reported as means ± standard deviation (SD). The results were evaluated by using Student's *t*-test and the Mann-Whitney rank-sum test, when appropriate. All statistical analyses were performed with SPSS Statistic 20 software (SPSS Inc., Chicago, IL). *P* values were considered significant when <0.05.

## 3. Results

### 3.1. Characterization of the Plasmatic Levels of Cytokines and Chemokines in CVD Patients and Potential Contribution of Venous Endothelium

In the first set of experiments, we sought to investigate the pattern of circulating cytokines/chemokines in CVD patients. For this purpose, plasma samples of CVD patients were collected before surgery for conservative and hemodynamic treatment of venous insufficiency in ambulatory care and analyzed for a panel of 31 cytokines/chemokines involved in the inflammatory and/or thrombosis processes. Overall, CVD patients (mean age of 52.3 ± 11.5) were characterized by a mean duration of the disease of 19.3 ± 11.6 years; besides 73% of them showed a family history of CVD with a variable incidence of other relevant comorbidities such as diabetes mellitus, hypertension, hypercholesterolemia, and cardiac disease (Supplementary Table 1). Among the 31 cytokines/chemokines analyzed by multiplex assay, 18 were detectable in plasma samples of both CVD patients and healthy control individuals: MIP-1*α*, IL-8, IL-7, IFN-*γ*, IL-12(p70), TNF-*α*, GM-CSF, IFN-*α*2, G-CSF, IL-1RA, MIP-1*β*, VEGF, EGF, Eotaxin, MCP-1, CXCL10, PDGF, and RANTES (Table 1). Of interest, within the panel of cytokines and chemokines detectable at the plasma level, 12 (MIP-1*α*, IL-8, TNF-*α*, GM-CSF, IFN-*α*2, MIP-1*β*, VEGF, EGF, Eotaxin, MCP-1, PDGF, and RANTES) were significantly (*P* < 0.05) increased in CVD patients compared to healthy controls while 2 additional cytokines (G-CSF and CXCL10) showed levels close to significance (Table 1). These data suggested the potential role of these cytokines/chemokines in the pathogenesis and/or progression of the disease.

To evaluate the role of pathological endothelium in the establishment of the increased levels of circulating cytokines/chemokines characterizing CVD, we have isolated pathological VEC from surgical specimens obtained from the same CVD patients analysed for the circulating cytokines/chemokines (Supplementary Table 1 and Table 1). VEC cultures were characterized and defined as CD31^+^/CD105^+^/CD146^+^/CD144^+^/CD45^−^/CD14^−^ cells, as previously described [9], and were assessed for the baseline release of the same panel of 31 cytokines/chemokines evaluated in plasma samples of CVD patients. In this respect, it should be noticed that since VEGF and EGF are essential components of the VEC culture medium, it was not possible to distinguish exogenously added VEGF and EGF from endogenously produced cytokines. Of interest, among the cytokines/chemokines detectable at the plasma of CVD patients (Table 1), only MIP-1*α* was undetectable in VEC culture supernatants (Table 2), while IL-8, IL-7, IFN-*γ*, IL-12(p70), TNF-*α*, GM-CSF, IFN-*α*2, G-CSF, IL-1RA, MIP-1*β*, Eotaxin, MCP-1, CXCL10, PDGF, and RANTES were released at different levels (Table 2). This group of data suggests that VEC represent a major cell type contributing to the production of plasmatic cytokines/chemokines in CVD patients.

**Figure 1:**
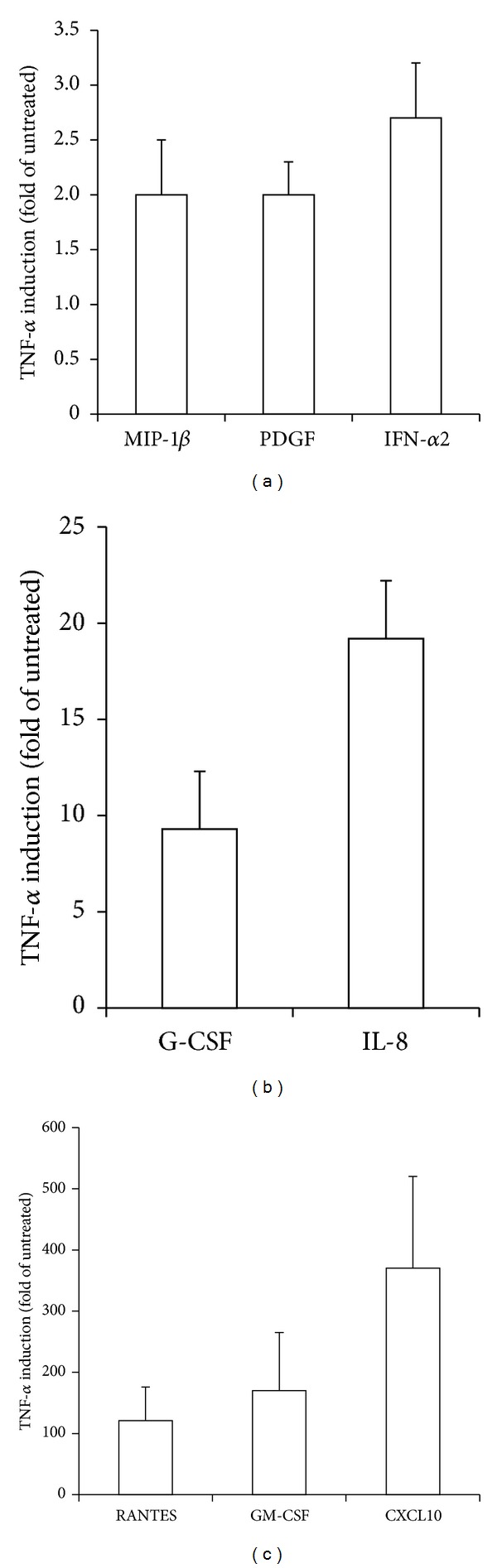
Cytokines/chemokines *in vitro* release by pathological VEC in response to TNF-*α*. Endothelial cells were exposed for 24 hours to TNF-*α* (5 ng/mL) before analysis of cytokines/chemokines release on culture supernatants. Results are expressed as fold of induction with respect to untreated cultures (set to 1). Only cytokines/chemokines showing fold of induction ≥2 have been reported. Data are means ± SD of results obtained in VEC from all CVD patients.

In light of the fact that pathological VEC are exposed *in vivo* to a proinflammatory *milieu *[7–11, 14] and that we found a significant (*P* < 0.01) elevation of the plasmatic levels of TNF-*α* in CVD patients, in further experiments VEC cultures were exposed *in vitro* to recombinant TNF-*α* in order to mimic the *in vivo* inflammatory microenvironment. Only those cytokines/chemokines showing a mean increase ≥2-fold were considered (Figure 1). These eight cytokines/chemokines were grouped on the basis of the fold increase: MIP-1*β*, PDGF, and IFN-*α*2 (between 2 and 3 mean fold increase), G-CSF and IL-8 (between 10 and 20 mean fold increase), and GM-CSF, CXCL10, and RANTES (between 100 and 300 mean fold increase) (Figure 1).

### 3.2. The Release of Specific Cytokines/Chemokines by Pathological VEC Is Differentially Inhibited by *α*-Lipoic Acid and Ginkgoselect Phytosome

**Figure 2:**
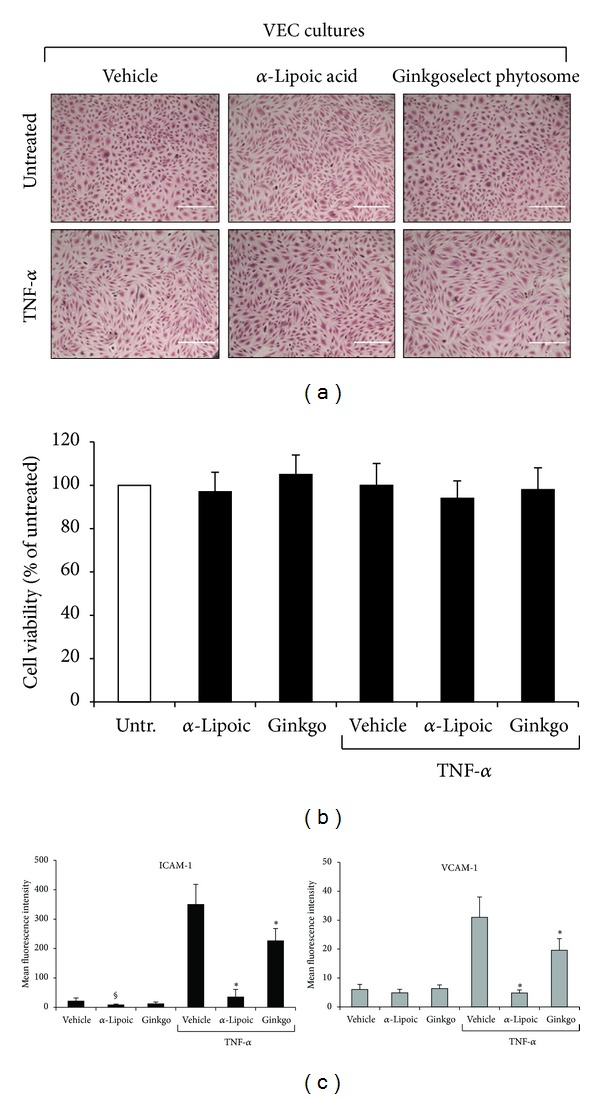
Lack of cell toxicity and anti-inflammatory properties of *α*-Lipoic acid and Ginkgoselect phytosome in CVD-VEC model. Endothelial cells were exposed to TNF-*α* (5 ng/mL), Ginkgoselect phytosome, and *α*-Lipoic acid (both at 100 *μ*g/mL) used either alone or in combination (1 hour of pretreatment with Ginkgoselect phytosome and *α*-Lipoic acid before addition of TNF-*α*) and analyzed after 24 hours of treatments. In (a), representative fields of cultures treated as indicated were observed by light microscopy after haematoxylin and eosin staining. In (b), cell viability is shown as percentage of untreated cultures (Untr.) set to 100%. Data are reported as means ± SD of three independent experiments. In (c), data are expressed as mean fluorescence intensity (MFI) after subtraction of background fluorescence from vehicle-treated cells. Results are reported as means ± SD of four independent experiments. *P < 0.05 compared to vehicle and TNF-*α* treated VEC; ^§^ *P* < 0.05 compared to vehicle treated VEC.

**Figure 3:**
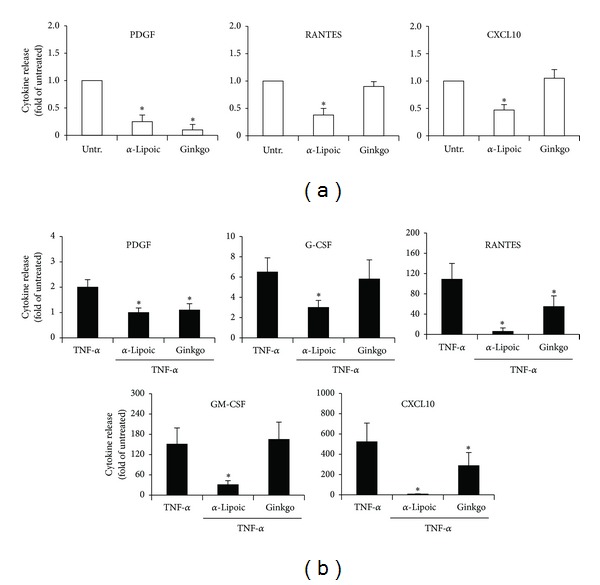
Inhibitory effects on cytokines/chemokines release of *α*-Lipoic acid and Ginkgoselect phytosome in pathological VEC cultures. Cytokines/chemokines levels were measured in culture supernatants of endothelial cells treated for 24 hours with *α*-Lipoic acid or Ginkgoselect phytosome used alone (a) or in combination with TNF-*α* (1 hour of pretreatment with *α*-Lipoic acid or Ginkgoselect phytosome before addition of TNF-*α*) (b). Results are expressed as fold of induction with respect to the untreated cultures (set to 1). Data are expressed as means ± SD. *P < 0.05 compared to untreated (a) or TNF-*α* treated cultures (b).

In the next set of experiments, we have investigated whether the anti-inflammatory natural compounds *α*-Lipoic acid [23–28] and *Ginkgo biloba* derivatives [29, 30] affected the release of cytokines/chemokines by pathological VEC. Indeed, although it has already been reported that both compounds, and in particular *α*-Lipoic acid, exhibit anti-inflammatory activities both *in vitro* and *in vivo* in the context of endothelial biology of different pathologies [23–27], it remains to be established whether these compounds are active also on pathological VEC isolated from CVD patients. Since both compounds have been shown to potentially affect the survival of endothelial cells [31–35], we started by analysing the morphology and viability of VEC exposed to different concentrations (up to 250 *μ*g/mL for each compound) of *α*-Lipoic acid and Ginkgoselect phytosome for 24 hours. As shown in Figure 2(a), morphological analysis did not reveal toxic effects at the concentration of 100 *μ*g/mL, when compounds were used either alone or together with recombinant TNF-*α*. The lack of toxicity indicated by morphological evidences was confirmed by quantitative cell viability analysis (Figure 2(b)). In addition, preexposure for 1 hour to both *α*-Lipoic acid and Ginkgoselect phytosome (both used at 100 *μ*g/mL), before addition of recombinant TNF-*α* for 24 hours, significantly (*P* < 0.05) inhibited the TNF-*α* mediated induction of the proinflammatory markers VCAM-1 and ICAM-1 (Figure 2(c)). Therefore, concentration of 100 *μ*g/mL for both compounds was selected for further experiments. Indeed, having confirmed that both *α*-Lipoic acid and Ginkgoselect phytosome exerted anti-inflammatory activities also in pathological VEC without inducing a specific toxicity (Figure 2), we analyzed the effect of both compounds on the specific release of cytokines/chemokines by pathological VEC. As shown in Figure 3(a), *in vitro* treatment with *α*-Lipoic acid alone was able to significantly decrease the baseline levels of PDGF, RANTES, and CXCL10, while Ginkgoselect phytosome alone only inhibited basal PDGF. In addition, pretreatment (for 1 hour) with both *α*-Lipoic acid and Ginkgoselect phytosome before addition of TNF-*α* significantly downregulated the release of TNF-*α*-induced PDGF, RANTES, and CXCL10 (Figure 3(b)), while *α*-Lipoic acid, but not Ginkgoselect phytosome, significantly inhibited the TNF-*α*-induced release of G-CSF and GM-CSF (Figure 3(b)). Thus, *α*-Lipoic acid exhibited a broader spectrum of inhibitory activity as compared to Ginkgoselect phytosome. On the other hand, the release of the other cytokines/chemokines upregulated by TNF-*α* (IFN-*α*2, MIP-1*β*, and IL-8) was unaffected by either *α*-Lipoic acid or Ginkgoselect phytosome (data not shown).

### 3.3. Different Intracellular Signaling Pathways Mediate the Cytokine/Chemokine Release by Pathological VEC

**Figure 4:**
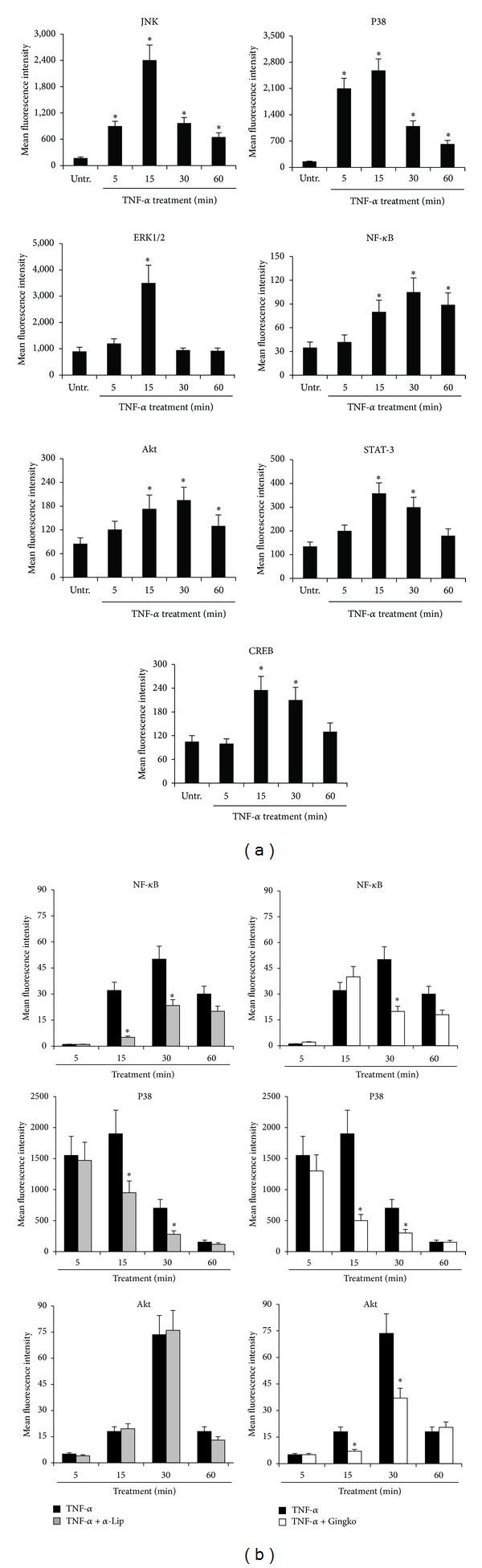
Multiplex analysis of molecular pathways activated in CVD-VEC in response to TNF-*α* stimulation: modulatory effect of *α*-Lipoic acid Ginkgoselect phytosome. Endothelial cells were left untreated (Untr.) or treated with TNF-*α* (5 ng/mL) alone (a) or in combination with *α*-Lipoic acid and Ginkgoselect phytosome (preincubation for 1 hour with *α*-Lipoic acid and Ginkgoselect phytosome both at 100 *μ*g/mL before addition of TNF-*α*; (b)) before analysis of phosphoprotein levels performed by Luminex system and expressed as mean fluorescent intensity (MFI). In (a), endothelial cells were treated with TNF-*α* for the indicated time points and cell lysates were assayed for phosphoproteins activation. *P < 0.05 compared to untreated cells. In (b), VEC cultures were pretreated for 1 hour with *α*-Lipoic acid and Ginkgoselect phytosome prior to stimulation with TNF-*α* for the indicated time points. Cell lysates were assayed for phosphoproteins induction and results expressed as means ± SD of samples assayed in duplicate in four independent experiments. *P < 0.05 compared to cells treated with TNF-*α* alone.

**Figure 5:**
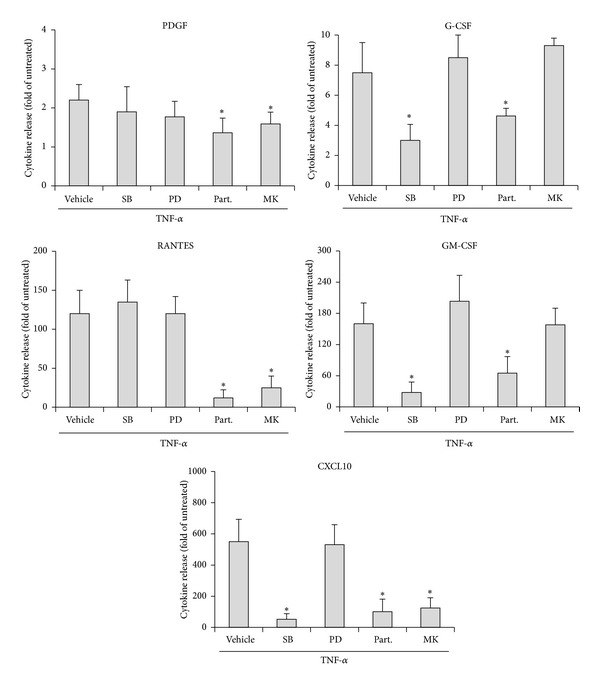
Differential effects of pharmacological inhibitors on cytokines/chemokines release in pathological VEC in response to TNF-*α*. VEC were pretreated for 1 hour with SB203580 (10 *μ*M), PD98059 (50 *μ*M), Parthenolide (10 *μ*M), SP600125 (5 *μ*M), and MK-2206 (5 *μ*M) before addition of TNF-*α* (5 ng/mL). After 24 hours, cell culture supernatants were analyzed for cytokines/chemokines release and normalized for cell number. Results are expressed as fold of induction with respect to untreated cultures (set to 1). Data are means ± SD of results obtained from four different experiments. *P < 0.05 compared to TNF-*α*/vehicle treated cells.

In the last group of experiments, we have investigated the intracellular signalling pathways mediating the release of the various cytokines and chemokines in the culture supernatants of pathological VEC and targeted by *α*-Lipoic acid and/or Ginkgoselect phytosome. In line with previous reports showing activation of various intracellular pathways by TNF-*α* treatment [36], we found that exposure of VEC cultures to recombinant TNF-*α* resulted in a time-dependent activation/phosphorylation of several signalling pathways such as JNK, p38/MAPK, ERK1/2, NF-*κ*B and Akt, STAT3, and CREB (Figure 4(a)). Of note, *α*-Lipoic acid significantly inhibited both NF-*κ*B and p38/MAPK pathways at two different time points (Figure 4(b)). On the other hand, Ginkgoselect phytosome significantly inhibited p38/MAPK and Akt pathways at two different time points, while it was less efficient than *α*-Lipoic acid in inhibiting NF-*κ*B. Neither of the two compounds was effective in modulating the remaining pathways analysed and shown in Figure 4(a). Based on the data illustrated above, the evidence that *α*-Lipoic acid was more powerful than Ginkgoselect phytosome in inhibiting the release of cytokines and chemokines, either spontaneously or in response to TNF-*α*, suggested that NF-*κ*B pathway plays a primary role in promoting the TNF-*α* mediated induction of cytokines and chemokines. To confirm this issue, in the last group of experiments, cells were preincubated with pharmacologic inhibitors specific for NF-*κ*B, p38/MAPK, Akt, and ERK1/2 pathways 1 hour before TNF-*α* addition. As shown in Figure 5, the release of PDGF, G-CSF, RANTES, GM-CSF, and CXCL10 was significantly reduced by Parthenolide, an inhibitor of NF-*κ*B pathway, which showed an overlapping pattern of inhibition with *α*-Lipoic acid (Figure 3), thus confirming the prominent role of NF-*κ*B in mediating the TNF-*α*-induced release of several cytokines/chemokines as well the role of NF-*κ*B as the major intracellular pathway targeted by *α*-Lipoic acid. Interestingly, the selective inhibitor of the p38/MAPK pathway SB203580 also significantly inhibited the release of G-CSF, GM-CSF, and CXCL10, suggesting that this pathway might also contribute to the release of cytokines/chemokines by VEC. Finally, the inhibitor of the Akt pathway induced a selective inhibition of PDGF, RANTES, and CXCL10 (Figure 5), showing a clear-cut overlapping with the inhibitory activity of Ginkgoselect phytosome (Figure 3). Consistently, Akt pathway represented a major target of Ginkgoselect phytosome (Figure 4(b)). The specificity of the different inhibitors of the pathways activated by TNF-*α* has been validated by Western blot, (see Supplementary Figure 1 in Supplementary Material available online).

## 4. Discussion

By analysing a large panel of cytokines/chemokines (*n* = 31), we demonstrated for the first time that patients affected by CVD are characterized by increased plasmatic levels of MIP-1*α*, IL-8, TNF-*α*, GM-CSF, IFN-*α*2, G-CSF, MIP-1*β*, VEGF, EGF, Eotaxin, MCP-1, CXCL10, PDGF, and RANTES with respect to healthy control subjects. Since the inflammatory properties of vein endothelium in CVD are poorly understood, an important point of our study was that all these cytokines, with the exception of MIP-1*α*, were spontaneously released *in vitro* by VEC purified from the same CVD patients analyzed for the plasmatic levels of cytokines/chemokines. An additional interesting finding was that the majority of the cytokines/chemokines found elevated in plasma of CVD patients and released spontaneously in the culture supernatants by VEC obtained from CVD patients (with the exception of VEGF and EGF that were not valuable since they were contained in the endothelial culture medium) were significantly induced by recombinant TNF-*α*, a major proinflammatory cytokine. Taken together, the *in vivo* and *in vitro* data suggested that a specific pattern of cytokines/chemokines (MIP-1*β*, PDGF, IFN-*α*2, G-CSF, IL-8, RANTES, GM-CSF, and CXCL10) (i) is produced by pathological VEC obtained from CVD patients, (ii) is significantly increased in response to TNF-*α*, and (iii) is elevated in the plasma of CVD patients. These findings suggest that pathological VEC significantly contribute to the elevation of the majority of plasmatic cytokines/chemokines in CVD although, obviously, the potential contribution of additional cellular sources to the production of plasmatic cytokines/chemokines is not excluded.

In order to evaluate potential anti-inflammatory compounds able to affect the release of this specific set of cytokines/chemokines, we have used *α*-Lipoic acid and *Ginkgo biloba* derivatives, taking into account previous studies, which have demonstrated that both compounds show anti-inflammatory activity on a variety of endothelial cell types [23–35], although they have never been tested before on pathological VEC. The anti-inflammatory activity of both *α*-Lipoic acid and *Ginkgo biloba* derivatives was confirmed on the basis of their ability to markedly inhibit the surface levels of ICAM-1 and VCAM-1, which are known to promote recruitment of leukocytes, platelets, and erythrocytes to the vein wall [37, 38], without inducing toxic effect of VEC cultures. We could demonstrate that, among the cytokines/chemokines found elevated in the plasma of CVD patients and whose *in vitro* release increased in response to recombinant TNF-*α*, *α*-Lipoic acid significantly (*P* < 0.05) decreased the basal release of PDGF, RANTES, and CXCL10 and the TNF-*α*-induced release of PDGF, RANTES, CXCL10, G-CSF, and GM-CSF. On the other hand, Ginkgoselect phytosome decreased the release only of basal PDGF and the TNF-*α*-induced PDGF, RANTES, and CXCL10. Thus, *α*-Lipoic acid showed a broader and more potent inhibitory activity on the release of cytokines/chemokines with respect to Ginkgoselect phytosome. Since recombinant TNF-*α* induced several intracellular pathways in VEC, such as the JNK, p38/MAPK, ERK1/2, NF-*κ*B, Akt, STAT3, and CREB pathways, it is noteworthy that *α*-Lipoic acid significantly inhibited TNF-*α*-induced NF-*κ*B and dampened p38/MAPK activation, while Ginkgoselect phytosome downmodulated TNF-*α*-induced p38/MAPK and Akt activation. Consistently with a prominent role of NF-*κ*B and to a lesser extent p38/MAPK pathway in promoting the release of a specific set of cytokines/chemokines in response to TNF-*α*, Parthenolide, a pharmacological inhibitor of the NF-*κ*B pathway, inhibited the release of PDGF, RANTES, CXCL10, G-CSF, and GM-CSF, recapitulating the effect of *α*-Lipoic acid, while MK-2206, a pharmacological inhibitor of the Akt pathway, inhibited the release of PDGF, RANTES, and CXCL10, recapitulating the effect of Ginkgoselect phytosome.

In conclusion, we have demonstrated that pathological VEC significantly contribute to the circulating cytokines/chemokines found elevated in plasma of CVD patients and by using both *α*-Lipoic acid and Ginkgoselect phytosome, we have characterized and targeted the prominent pathways promoting the release of these cytokines and chemokines.

## Supplementary Material

Supplementary Table 1: Main demographic and clinical characteristics of CVD patients involved in the study.
Supplementary Figure 1: Western blotting validation of the specificity of the different inhibitors of the intracellular pathways activated by TNF-*α*.

## Figures and Tables

**Table 1:** Circulating levels of cytokines/chemokines and growth factors in healthy controls and CVD patients.

Cytokines/Chemokines	Plasma levels (pg/mL)^a^		Limit of detection (pg/mL)
	Controls	CVD patients	
IL-10	<OOR	<OOR	
IL-12 (p40)	<OOR	<OOR	1.1
IL-13	<OOR	<OOR	7.4
IL-15	<OOR	<OOR	1.3
IL-17A	<OOR	<OOR	1.2
IL-1*α*	<OOR	<OOR	0.7
IL-1*β*	<OOR	<OOR	9.4
IL-2	<OOR	<OOR	0.8
IL-3	<OOR	<OOR	1.0
IL-4	<OOR	<OOR	0.7
IL-5	<OOR	<OOR	4.5
IL-6	<OOR	<OOR	0.5
TNF-*β*	<OOR	<OOR	0.9
MIP-1*α*	0 (0.87 ± 2)	13 (13.7 ± 14.5)**	1.5
IL-8	2.2 (2.8 ± 1.8)	14.4 (25.8 ± 35.5)*	2.9
IL-7	2.7 (2.9 ± 1.3)	3.7 (3.5 ± 1.3)	0.4
IFN-*γ*	3.18 (3.7 ± 1.9)	2.5 (4.4 ± 3.8)	1.4
IL-12 (p70)	3.2 (3.8 ± 2)	3.5 (3.7 ± 1.3)	0.8
TNF-*α*	3.3 (3.8 ± 1.6)	6.9 (6.8 ± 3.3)**	0.6
GM-CSF	4.6 (4.9 ± 1.9)	5.9 (6.7 ± 3)*	0.7
IFN-*α*2	9.5 (12 ± 8.3)	15 (17.7 ± 7.3)*	7.5
G-CSF	16.9 (17.9 ± 6.8)	29.2 (28.1 ± 18.1)^§^	2.9
IL-1RA	18.3 (20.3 ± 10)	16.7 (20.2 ± 11.5)	1.8
MIP-1*β*	19.6 (20.2 ± 6.8)	50 (54.5 ± 35.7)**	8.3
VEGF	29.2 (34.8 ± 17)	64.6 (63.4 ± 33.5)**	3.0
EGF	36.6 (40 ± 23.3)	95.4 (155.5 ± 152.5)*	26.3
Eotaxin	48.6 (54.9 ± 26)	251 (260 ± 211.7)**	2.8
MCP-1	161 (162.5 ± 56)	224 (276.2 ± 146.5)*	4.0
CXCL10	195 (203.5 ± 67.8)	244 (312.5 ± 178.9)^§^	1.9
PDGF	2,770 (5,476 ± 5,027)	9,369 (17,352 ± 20,163)*	8.6
RANTES	10,414 (16,126 ± 15,567)	45,399 (58,283 ± 59,353)*	2.2

^a^Values are expressed as median (mean ± SD); <OOR: out (below) of detection range. **P < 0.01; *P < 0.05; ^§^ *P* < 0.06.

**Table 2:** Baseline release of cytokines/chemokines and growth factors by pathological VEC.

Cytokines/Chemokines	Release levels (pg/mL/24 hours)^a^	Limit of detection (pg/mL)
MIP-1*α*	<OOR	2.9
IL-8	2,616 (3,111.8 ± 2,532.4)	0.4
IL-7	11.6 (16.7 ± 10.1)	1.4
IFN-*γ*	2.1 (2.3 ± 0.9)	0.8
IL-12 (p70)	2.8 (2.8 ± 0.4)	0.6
TNF-*α*	1.8 (1.6 ± 0.3)	0.7
GM-CSF	8.2 (8.6 ± 5.5)	7.5
IFN-*α*2	9.1 (9.3 ± 3.7)	2.9
G-CSF	49 (65.9 ± 37)	1.8
IL-1RA	10.4 (11.4 ± 4.2)	8.3
MIP-1*β*	4 (4.5 ± 2.1)	3.0
VEGF	ND	26.3
EGF	ND	2.8
Eotaxin	10.2 (15.1 ± 8.5)	4.0
MCP-1	8,663 (8,630.1 ± 1,450)	1.9
CXCL10	20.5 (41.6 ± 55)	8.6
PDGF	1,399 (1,494.6 ± 468.9)	2.2
RANTES	31 (33.3 ± 14.9)	1.2

^a^Values are expressed as median (mean ± SD); <OOR: out (below) of detection range; ND: not determinable because component of the endothelial medium.
